# Pharmacological effects of caffeine on ventilation in adult zebrafish under free-swimming conditions

**DOI:** 10.1038/s41598-022-22681-4

**Published:** 2022-10-21

**Authors:** Yuki Harada, Zu Soh, Shin Wakitani, Masayuki Yoshida, Toshio Tsuji

**Affiliations:** 1 Graduate School of Advanced Science and Engineering, Hiroshima University, Higashi-Hiroshima, 739-8527 Japan; 2 Graduate School of Integrated Sciences for Life, Hiroshima University, Higashi-Hiroshima, 739-8528 Japan

**Keywords:** Behavioural methods, Predictive markers, Signal processing

## Abstract

The zebrafish is widely used as a model in biological studies. In particular, the heart rate and cortisol levels of zebrafish are commonly measured to elucidate the pharmacological effects of chemical substances. Meanwhile, although ventilation is also an important physiological index reflecting emotion-like states, few studies have evaluated the effects of chemicals on ventilation in adult zebrafish. In this study, we assessed whether it is possible to evaluate the pharmacological effects elicited by caffeine in adult zebrafish under free-swimming conditions. We measured the ventilation in adult zebrafish exposed to multiple concentrations of caffeine under restraint and free-swimming conditions and evaluated the pharmacological effects of caffeine using linear mixed model analysis. In addition, results of electrocardiogram analysis and swimming speeds were compared with those in previous reports to ensure that an appropriate dose of caffeine was administered. Under restraint conditions, caffeine significantly decreased heart rate and increased ventilation in a concentration-dependent manner. Under free-swimming conditions, the ventilation rate significantly increased with increasing caffeine concentration. These results indicate that the pharmacological effects elicited by chemicals on ventilation can be evaluated in free-swimming zebrafish.

## Introduction

Zebrafish are commonly employed as an animal model for biological studies as their genome sequence has been reported^1^ and their organ structures and functions are similar to those of humans^2–5^. In particular, their transparent bodies have made embryos and larvae convenient for use in various studies, including those related to drug discovery^6–8^ as direct observation and manipulation of organs and cells is possible^9^. However, the autonomic nervous system^10,11^ and gill ventilation^12–14^ are not fully functional in the larval stage; hence, the pharmacological effects exerted on these functions cannot be assessed until later stages.

Zebrafish are commonly used as a human heart disease model^15–18^. In fact, cardiac activity is the most common physiological index assessed in adult zebrafish^19,20^. The heart rate of adult zebrafish is influenced by autonomic nervous system activity, and the cardiac action potential is similar to that of humans. As such, the autonomic activity of the zebrafish heart is often assessed by measuring heart rate, and the associated variability, as in humans^19^. However, given that cardiac potential measurements require insertion of needle electrodes, spontaneous cardiac responses are not readily captured due to stress from restraint and nociception.

Ventilation is also an important physiological index and is closely related to the emotion-like state of fish. That is, when zebrafish encounter a new environment, they express anxiety-like behavior, and their ventilatory rate increases^21^. Subsequently, along with habituation and attenuation of anxiety-like behaviors, the ventilatory rate decreases and the variability of the ventilatory interval increases^22^. Importantly, adult fish generate bioelectrical signals synchronized with ventilation (ventilatory signals) that can be measured without direct contact using electrodes placed outside the body. More specifically, ventilatory signals have been measured using electrodes placed inside a small chamber in which the fish are loosely restrained^23–25^. Meanwhile, our research group has developed a system that can measure ventilatory signals under free-swimming conditions by placing electrode arrays at the bottom of the measurement tank^26^, facilitating the successful simultaneous measurement of movement and ventilation^27,28^. However, the response of zebrafish ventilation to chemical substances remains largely unknown.

Caffeine elicits both stimulant and anxiogenic pharmacological effects depending on its concentration^29^. Similar behavioral responses have been observed in zebrafish, when exposed to low concentrations of caffeine, fish exhibit leadership behavior, while they develop anxiety/fear-like states in response to high concentrations of caffeine^30^. Physiological responses have also been evaluated by measuring heart rate and cortisol (a stress-sensitive hormone) levels^31^. Exposure to high concentrations of caffeine significantly decreases the heart rate^32^ and increases stress-sensitive cortisol levels^31^. Hence, given that caffeine affects the behavior and physiological state of zebrafish, it may also affect ventilation.

In this study, we assessed the feasibility of evaluating the pharmacological effects of caffeine in zebrafish under free-swimming conditions. To this end, we exposed zebrafish to multiple concentrations of caffeine under restraint and free-swimming conditions. Under restraint, an electrocardiogram (ECG) was obtained by inserting needle electrodes, and ventilation was measured by recording gill movements with a high-speed camera. The heart rate and heart rate variability were obtained from the ECG. In addition, the ventilatory frequency and coefficient of variation of the ventilatory interval were obtained from gill movements. We then used a linear mixed model to evaluate the effect of caffeine on cardiac activity under restraint and examined whether the results were consistent with previously reported trends^32^. Furthermore, we measured ventilation signals under free-swimming conditions using a measurement system developed by our research group and analyzed ventilatory indices using a linear mixed model to compare the ventilatory responses under free-swimming and restraint conditions.

## Results

### Restraint conditions

**Figure 1:**
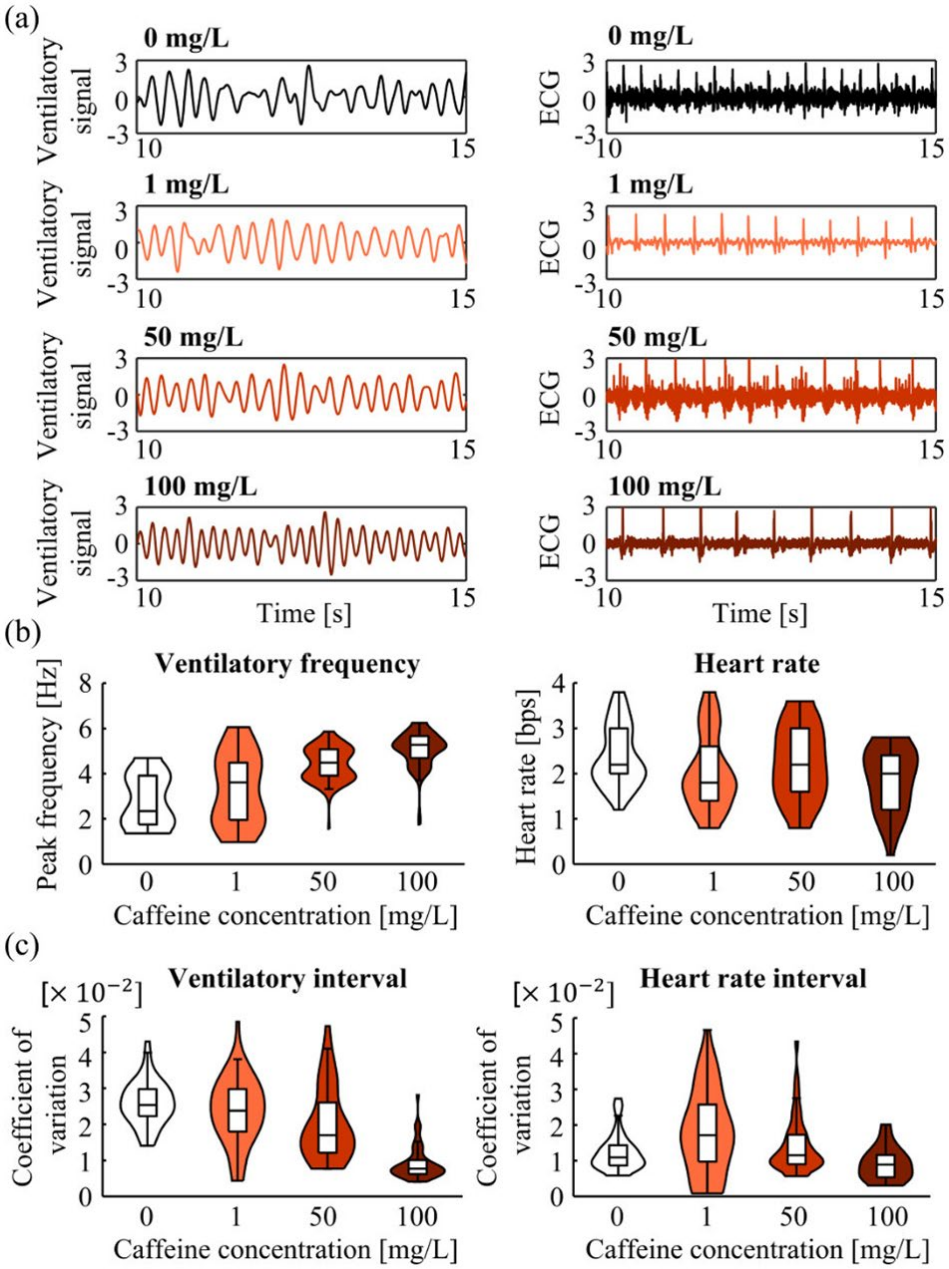
Frequency and variability analyses under restraint. Data were obtained from five individuals per caffeine concentration except for the 50 mg/L dose, for which four individuals were included as one did not meet the data inclusion criterion of surviving 72 h after the experiment. Violin plots of 0, 1, and 100 mg/L doses included 120 (five individuals, 120 s of measurement duration divided into 5 s segments) data points each; that of the 50 mg/L dose included 96 data points. The boxplots in the violin plot present the upper quartile, median, and lower quartile; whiskers extend from maximum to minimum value, excluding outliers. (**a**) Standardized ventilatory signals and ECG. (**b**) Distribution of ventilatory peak frequencies and heart rate at each caffeine concentration. (**c**) Distribution of the coefficients of variation calculated from the ventilatory signals and ECG at each caffeine concentration.

Restrained adult fish (approximately 6 months of age and 35 mm in length) were exposed to caffeine solutions at concentrations of $x_c = 0, 1, 50$, and 100 mg/L; ECG and ventilation were measured for 120 s. The time-series data were divided into 5 s segments; frequency analysis was performed for each segment. Five individuals were monitored for each concentration (Supplementary Information). Fish that died within 72 h of completing the experiment were not included in the analysis. Accordingly, one fish from the 50 mg/L caffeine group was excluded. Figure 1a presents the waveform of one 5 s segment measured for each concentration, demonstrating the periodic ventilatory signals and ECG results. Figure 1b shows the violin plots indicating the distribution of the ventilatory peak frequency and heart rate for five individuals (data points of five individuals $\times$ 24 segments = 120 segments). The peak ventilatory frequency and heart rate were widely distributed between 1 and 6 Hz and 1–4 bps, respectively, indicating large variations among individuals. Intra- and inter-individual differences were controlled using a linear mixed model to examine the fixed effects of caffeine concentrations, as shown in the following equation:

$$\begin{aligned} F_j = \beta _1C+ \left( \beta _0+\beta _{0,C}+\beta _{0,C,j}\right) , \end{aligned}$$

where $F_j$ is the peak ventilatory frequency or heart rate of the individual $j=1,2,\ldots ,20$, $C=\log (x_c+1)$ is the logarithmic caffeine concentration, $\beta _0$ and $\beta _1$ are the fixed effects of the intercept and slope, respectively, $\beta _{0,C}$ is the random effect of the intercept depending on the concentration, and $\beta _{0,C,j}$ is the random effect of the intercept depending on the concentration and individual. This model was selected from equations (Eqs. 1–8) based on Akaike’s Information Criterion (AIC). The results are presented in Tables 1 and 2, respectively. Caffeine concentration had a significant fixed effect, $\beta _1$, on peak ventilatory frequency and heart rate ($p<0.05$). $\beta _1$ of caffeine concentration on peak ventilatory frequency and heart rate was positive and negative, respectively, indicating that the ventilatory frequency increased and heart rate decreased with increasing caffeine concentration. This trend in heart rate is consistent with a previous report^32^.

We then calculated the coefficients of variation for ventilatory interval and heartbeat. The violin plot in Fig. 1c shows the distribution of the coefficients of variation calculated from the 120 segments (5 s per segment) of ventilatory signals and ECG of five individuals measured at each concentration. The coefficients of variation for the ventilatory intervals were analyzed using a linear mixed model, and the results are shown in Table 1. A significant negative $\beta _1$ was observed ($p<0.05$), indicating that the ventilatory interval coefficient of variation decreased as caffeine concentration increased. Meanwhile, the heartbeat coefficient of variation increased with caffeine concentration between 0 and 1 mg/L and showed a decreasing trend from 50 to 100 mg/L (Fig. 1c). Therefore, the analysis was divided into two sections: 0–1 mg/L and 1–100 mg/L. The parameters of the linear mixed model obtained for both sections are listed in Table 2. Significant $\beta _1$ values were detected in both sections ($p<0.05$), indicating that the heartbeat coefficient of variation increased at low caffeine concentrations and decreased at high concentrations, which may reflect the stimulant and anxiogenic effects reported in previous studies^29,30^.

### Free-swimming conditions

To examine the effects of caffeine under free-swimming conditions, the bioelectrical signals generated via ventilation were measured using 126 electrodes placed at the bottom of the tank in our previously developed measurement system^26,28^. The signals were recorded for 120 s and five individuals for each concentration. The recorded time-series data were equally divided into 5 s segments for analysis, similar to the restraint conditions. Note that the measured bioelectrical signals represented gill movements (Supplementary Information S3). Furthermore, to verify the validity of the experiment, we measured the motion of the fish using a video camera. The position of the fish was then tracked using a motion-tracking software DeepLabcut^33^, and the average swimming speed of each 5 s interval was calculated.

**Figure 2:**
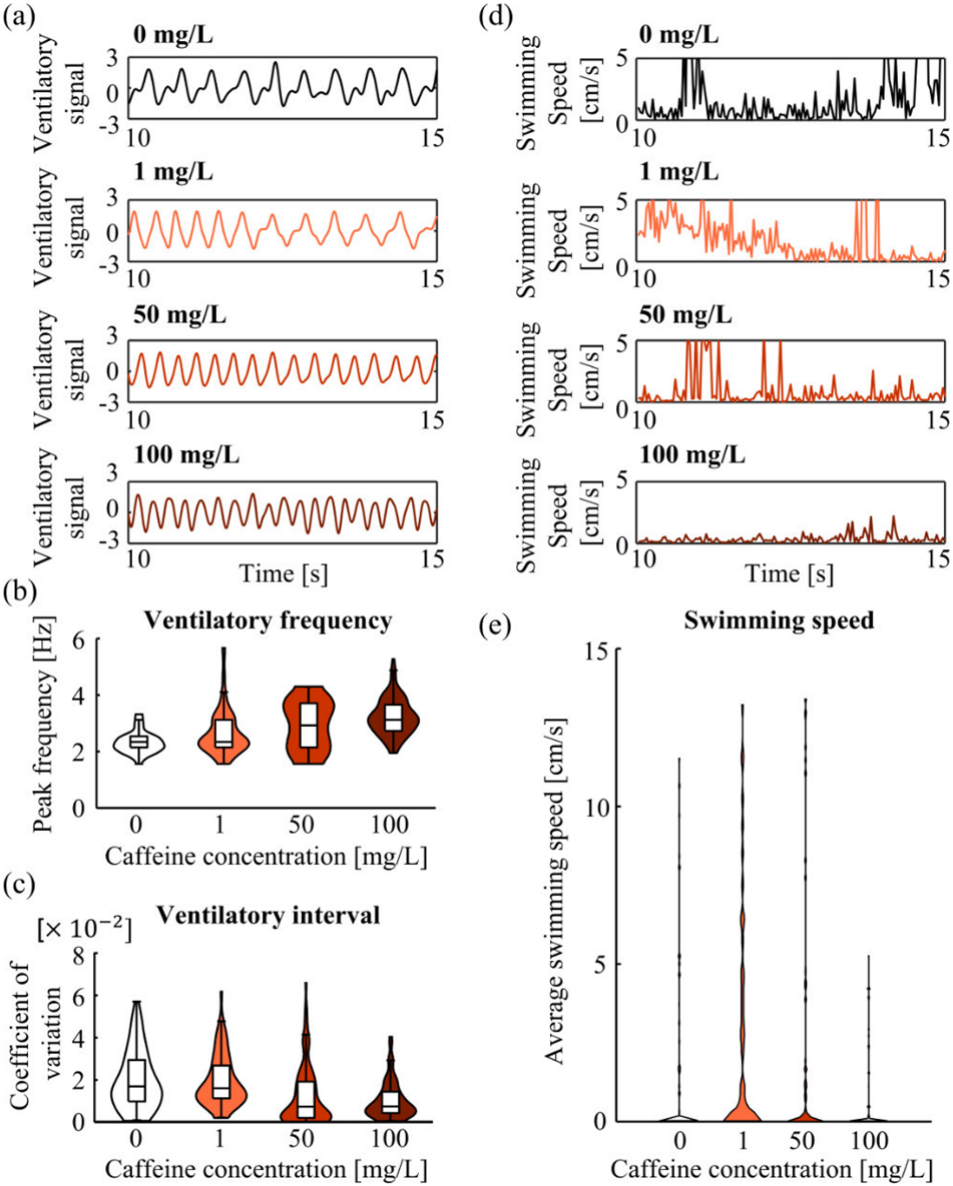
Peak frequencies of ventilatory signals, ventilation variability, and swimming speeds under free-swimming conditions. Data were obtained from five individuals per caffeine concentration. Violin plots include 120 (five individuals, 120 s of measurement duration divided into 5 s segments) data points each. The boxplots in the violin plot present the upper quartile, median, and lower quartile, and whiskers extend from maximum to minimum value, excluding outliers. (**a**) Standardized ventilatory signals. (**b**) Distribution of peak frequencies for ventilatory signals at each caffeine concentration. (**c**) Distribution of coefficients of variation for ventilatory intervals at each caffeine concentration. (**d**) Swimming speed waveform. (**e**) Distribution of average swimming speeds of each 5 s interval at each caffeine concentration.

Periodic ventilatory signals was also measured under free-swimming conditions (Fig. 2a). Caffeine concentration had a significant $\beta _1$ on both the peak ventilatory frequency and coefficient of variation of the ventilatory interval ($p<0.05$) (Fig. 2b, c and Table 1). Hence, as the caffeine concentration increased, the ventilatory frequency increased, and the ventilatory interval coefficient of variation decreased, consistent with the results obtained under restraint conditions. The average swimming speed increased with increasing caffeine concentration between 0 and 1 mg/L while exhibiting a decreasing trend from 50 to 100 mg/L (Fig. 2d, e, Table 1). Therefore, similar to that performed for the restrained conditions, the analysis was divided into two sections: 0–1 mg/L and 1–100 mg/L (Table 3). The $\beta _1$ was significant for caffeine concentration in the 1–100 mg/L section($p<0.05$), but not in the 0 to 1 mg/L section. Thus, the average swimming speed decreased at high concentrations of caffeine, which is consistent with results of a previous report^30^.

**Table 1:** Linear mixed model analysis for ventilation indices.

Parameter name	Restraint conditions		Free-swimming conditions	
	Peak frequency	Coefficient of variation	Peak frequency	Coefficient of variation
$\beta _1$ (slope)	$3.33 \times 10^{-1}$	$-2.93\times 10^{-2}$	$1.60 \times 10^{-1}$	$-1.44 \times 10^{-3}$
*p* value of $\beta _1$	**$1.38 \times 10^{-3}$	***$3.19 \times 10^{-4}$	**$8.47 \times 10^{-3}$	*$3.83 \times 10^{-2}$
Confidence interval of $\beta _1$	$[0.791, 5.87] \times 10^{-1}$	$[-4.30, -1.54] \times 10^{-2}$	$[0.470, 2.72]\times 10^{-1}$	$[-\,2.77, -\,0.103]\times 10^{-3}$
$\beta _0$ (intercept)	3.36	$2.62\times 10^{-1}$	2.41	$1.74\times 10^{-2}$
*p* value of $\beta _0$	***$2.05 \times 10^{-8}$	***$5.73 \times 10^{-11}$	***$4.62 \times 10^{-12}$	***$2.43 \times 10^{-8}$
Confidence interval of $\beta _0$	[2.58, 4.14]	$[2.20, 3.03] \times 10^{-1}$	[2.06, 2.75]	$[1.33, 2.15]\times 10^{-2}$
Standard deviation of random effects	0.384	0.0292	0.0409	$8.96 \times 10^{-3}$

20 individuals were used for the restraint and free-swimming experiments. An individual included in the restraint experiment in the 50 mg/L dose group was excluded from analysis. (*:$p<0.05$, **:$p<0.001$, ***:$p<0.001$).

**Table 2:** Linear mixed model analysis for heart rate indices under restraint conditions.

Parameter name	Heart rate	Coefficient of variation (0–1 mg/L)	Coefficient of variation (1–100 mg/L)
$\beta _1$ (slope)	$-5.29\times 10^{-2}$	$7.44\times 10^{-2}$	$-7.56 \times 10^{-2}$
*p* value of $\beta _1$	*$2.29 \times 10^{-2}$	*$3.62 \times 10^{-2}$	***$1.05 \times 10^{-6}$
Confidence interval of $\beta _1$	$[-8.68, -1.90]\times 10^{-2}$	$[2.48, 1.24]\times 10^{-2}$	$[-\,1.12, -\,0.326]\times 10^{-1}$
$\beta _0$ (intercept)	2.26	$1.24\times 10^{-1}$	$4.28\times 10^{-1}$
*p* value of $\beta _0$	***$2.28 \times 10^{-9}$	***$2.00 \times 10^{-16}$	***$1.87 \times 10^{-9}$
Confidence interval of $\beta _0$	[2.15, 2.36]	$[0.999, 1.49]\times 10^-2$	$[3.11, 5.48]\times 10^{-1}$
Standard deviation of random effects	$6.43\times 10^{-1}$	$7.38 \times 10^{-2}$	$2.47\times 10^{-1}$

(*:$$p<0.05$$, **:$$p<0.001$$, ***:$$p<0.001$$).

**Table 3:** Linear mixed model analysis for average swimming speed under free-swimming conditions.

Parameter name	Average swimming speed (0–1 mg/L)	Average swimming speed (1–100 mg/L)
$\beta _1$ (slope)	19.6	$-6.04$
*p* value of $\beta _1$	$2.47\times 10^{-1}$	**$9.91 \times 10^{-3}$
Confidence interval of $\beta _1$	$[-\,16.1, 54.6]$	$[-\,10.1, -\,1.99]$
$\beta _0$ (intercept)	10.1	27.7
*p* value of $\beta _0$	$2.27 \times 10^{-1}$	$1.78 \times 10^{-3}$
Confidence interval of $\beta _0$	[7.06, 27.7]	[14.2, 41.3]
Standard deviation of random effects	11.8	7.53

(*:$$p<0.05$$, **:$$p<0.001$$, ***:$$p<0.001$$).

## Discussion

This study aimed to assess the feasibility of measuring the ventilatory response of zebrafish to caffeine under free-swimming conditions. It was first necessary to confirm that responses to caffeine at different doses were appropriately evoked. To this end, we measured ECG and ventilation under restraint and swimming motion under free-swimming condition. We then confirmed that the cardiac activity and motor response to caffeine were consistent with previously reported findings^30,32^. The results showed consistent ventilatory response trends under restraint and free-swimming conditions, and thus, the ventilatory responses measured under free-swimming conditions were deemed reasonable.

Caffeine has stimulating effects at low concentrations and anxiogenic effects at high concentrations^30,34^. In the current study, under restraint, heart rate decreased with increasing caffeine concentration (Fig. 1c), which agreed with previous larvae results^32^, confirming that the caffeine stimuli were appropriately applied. Moreover, the analytic results of ventilation indicated that caffeine dose increased ventilatory rate and decreased coefficients of variation for the ventilatory intervals. These observations were consistent with the effects of caffeine dose in humans^35^. In the case of humans, this response is induced by caffeine stimulating the central vagus nerve and respiratory center of the medulla oblongata^35^; however, it remains unclear whether the same mechanism is employed in zebrafish.

Under free-swimming conditions, although there was no significant change in the average swimming speed under low caffeine exposure, an increasing trend was observed (Fig. 2e). In addition, a high concentration of caffeine exposure significantly decreased swimming speed. These trends were consistent with those reported in previous studies^30,36^ evaluating the behavior of zebrafish during caffeine exposure and may reflect the stimulating effect of exposure to low-concentration and the anxiogenic effect of exposure to high-concentration. Therefore, we inferred that appropriate caffeine stimuli were also applied under free-swimming conditions. However, there was a difference in the threshold concentration at which swimming speed began to decrease. While literature reports a significant decrease in swimming speed at 50–150 mg/L compared with that at 10 and 25 mg/L doses^36^, our results showed a significant decrease in swimming speed at 50–100 mg/L compared with that at 1 mg/L, indicating that the threshold for decreased swimming speed shifted to low concentrations. The cause of this shift is unclear and warrants further investigation by extending measurement duration or acclimating the fish to a tank in advance.

Under both restraint and free-swimming conditions, caffeine significantly increased the ventilatory frequency and decreased the coefficient of variation of ventilatory intervals (Table 1). We compared the confidence intervals of the fixed effects of the slope, $\beta _1$, and the intercept, $\beta _0$, for the restraint and free-swimming conditions and found that they did not overlap and the fixed effects were smaller under free-swimming conditions. The difference in the fixed effects between the restraint and free-swimming conditions may be due to stress induced by restraint and nociception. The standard deviation of the random effect expressed by the terms $\beta _{0, C}$ and $\beta _{0, C,j}$ was smaller under free-swimming conditions than under restraint. The increase in standard deviation might be due to individual differences in response to anesthesia and restraint. These findings highlight the effectiveness of analysis under free-swimming conditions, which can minimize individual differences.

The experimental environment and protocol of this study limited the ability to filter the effects of parameters other than caffeine. That is the obtained data involved effects of anesthesia and nociception under restraint conditions, and anxiogenic response to the novel environment and shallow water level under free-swimming conditions. According to previous studies, 2-phenoxyethanol is toxic to fish sized 30 ± 5 mm upon long-term exposure (96 h) at a high concentration (330 mg/L)^37,38^. However, the exposure duration was markedly shorter in the current study, and all individuals recovered from anesthesia after the experiments. However, one individual did not survive for 72 h after completion of the study, likely due to the deep needle electrode insertion. Therefore, we considered the exposure duration and concentrations acceptable. Nevertheless, the exposure could suppress ventilation and cardiac activity^39^, and thus the analyzed data included the effects of anesthesia. The experiment under free-swimming conditions was performed according to a previously described experimental protocol^5^, in which individuals in the experimental group were transferred from the preparation tank to the measurement tank. Our previous novel tank tests under free-swimming conditions indicated a rapid attenuation of the initial fear/anxiety-like responses to a novel environment over the first 10 min, and the ventilatory frequency significantly decreased every 3 min^22^. The mean ventilatory frequency for the first 3 min was approximately 4.00 Hz, which decreased to 2.75 Hz after 1 h of habituation. For the free-swimming protocol, the 3 min before measurement was intended to reduce the acute response to the transfer, which, according to this previous study, was insufficient to minimize the effect of habituation^22^. In addition, the height of the water column of the measurement tank was as shallow as 30 mm, which was set to measure the electrical ventilatory signals, however, may also evoke anxiety-like behavior^31^. The measured data thus includes these anxiogenic and habituation effects under free-swimming conditions. Further, the measurement time was shortened given that stress levels were expected to increase under restraint. Therefore, the measured heart rate and ventilation might only reflect transient responses to caffeine. In addition, due to the small *n* value, this study failed to account for sex- and genotype-dependent differences. Indeed, a previous nicotine administration study indicated a significant sex-based effect^40^; it is highly possible that sex differences also affect the response to caffeine^41^. Moreover, genotype-dependent responses are not a trivial issue^31^, which limited direct comparison of our experimental results with previous publications^30–32,42^. Nevertheless, these issues will be addressed in future studies.

This study elucidated consistencies and inconsistencies in the ventilatory response to caffeine between restraint and free-swimming conditions. Specifically, a consistent ventilatory response was observed, while a smaller effect size under free-swimming conditions compared to restraint conditions. Hence, application of a free-swimming model, in place of a restrained model, to assess pharmacological effects elicited by chemicals will prove beneficial. Additionally, experiments under free-swimming conditions may provide novel insights regarding the relationship between ventilatory and motor responses. For example, it is considered that ventilatory information represents an equally important biomarker as motor information for emotion-like behaviors^21^, however, currently, ventilatory information is scarcely used together with motor responses to assess emotion-like behaviors. In addition, although ventilatory signals have been measured to assess the respiratory response to hypoxia, hyperoxia, and hypercapnia^23–25^, the associated motor response was not assessed. Therefore, conducting these experiments under free-swimming conditions can provide a deeper understanding of the emotion-like behavior and response to water gas composition. The free-swimming experiment can also be applied to assess the effect of caffeine on habituation, which we could not fully address in the current study. In this way, the free-swimming assay can be applied to the field of drug discovery.

## Materials and methods

### Animals

Forty adult zebrafish (*Danio rerio*) (approximately 6 months old) (Table 4)with an AB genetic background^43^ and average body length of approximately $37 \pm 3$ mm were housed in groups of 5–7 and placed in 2 L water tanks at a temperature between 26 $^{\circ }$C and 28 $^{\circ }$C with a 14:10 h light:dark cycle. All procedures were performed in accordance with the ethical guidelines for animal experimentation set out by Hiroshima University, and our protocol was approved by the Committee for Animal Experimentation at Hiroshima University, Japan (approval number F19-1). All methods are reported in accordance with ARRIVE guidelines for the reporting of animal experiments. Table 4 lists the number and sex of fish recruited in each experimental condition.

**Table 4:** Number of individuals.

Concentrations (mg/L)	Restraint	Free-swimming
0	5 (M:2, F:3) $\times$ 24 seg. = 120	5 (M:4, F:1) $\times$ 24 seg. = 120
1	5 (M:2, F:3) $\times$ 24 seg. = 120	5 (M:4, F:1) $\times$ 24 seg. = 120
50	4 (M:2, F:2) $\times$ 24 seg. = 96	5 (M:3, F:2) $\times$ 24 seg. = 120
100	5 (M:2, F:3) $\times$ 24 seg. = 120	5 (M:2, F:3) $\times$ 24 seg. = 120

seg., segments; M, male; F, female.

### Experiments under restraint

#### The measurement system

**Figure 3:**
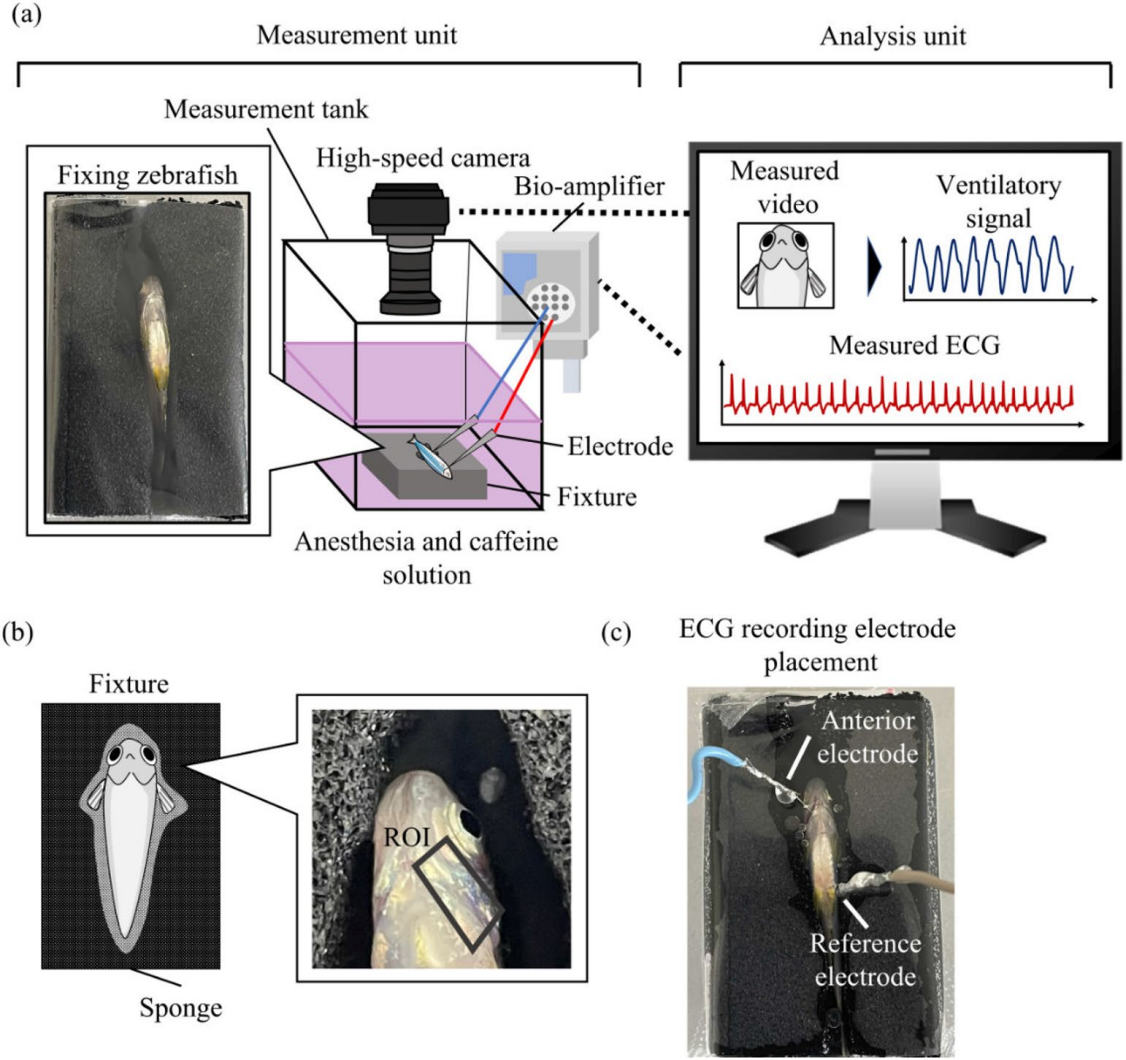
Measurement equipment for experiment under restraint conditions. (**a**) Schematic diagram of measurement and analysis units. (**b**) Region of interest (ROI) around the gill. (**c**) Electrode insertion into the fish.

The measurement system comprised a fixture, measurement tank, high-speed camera, bioamplifier, and needle electrodes (Fig. 3a). The fixture was made up of a conductive urethane sponge hollowed in the shape of a fish and wrapped in transparent vinyl to hold the fish in place. The fixture was attached to the bottom of the measurement tank using hook and loop fasteners, and two needle electrodes were inserted into the fish through the vinyl to measure the ECG. The measured ECG was sampled at $f_s$ Hz using a digital bioamplifier (EEG-1200, Nihon Kohden, Tokyo, Japan).

To measure ventilation and capture images of the zebrafish head at $f_c$ fps, a high-speed camera (Ace USB3.0, Basler AG, Ahrensburg, Germany) was installed above the measurement tank. Then, polygonal regions of interest (ROIs) were set around the gill area of the captured video image, as shown in Fig. 3b to detect the gill movement. Specifically, the mean brightness in the ROI was calculated for each frame, and the obtained time-series waveform was defined as the ventilatory signal.

#### Experimental protocol

**Figure 4:**
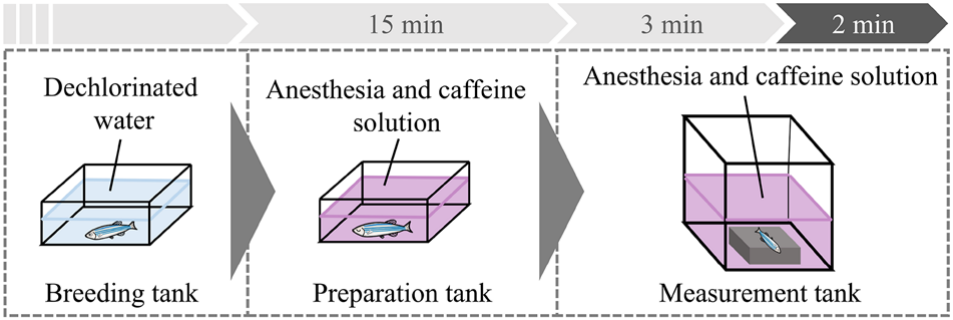
Experimental protocol of the restraint conditions.

The experimental procedure is illustrated in Fig. 4. A mixture of dechlorinated water, 2-phenoxyethanol (for anesthesia), and caffeine were prepared. Exactly, 400 mL of the mixture, unlike MS-222 and Eugenol, was added to both the preparation and measurement tanks. The concentrations of 2-phenoxyethanol and caffeine were $x_a$ $\upmu$L/L and $x_c$ mg/L, respectively. 2-phenoxyethanol was used as it does not cause loss of ventilation, unlike MS-222 and Eugenol^39^. Given that there were six breeding aquariums, before each experiment we rolled a dice to determine the breeding aquarium from which a fish would be captured. The order of experimental conditions to be performed was predetermined using the random number generator in MATLAB 2021, and the experimenter was blinded to the experimental conditions before capturing an individual. The randomly chosen individual was transferred from the breeding tank to the preparation tank and exposed to 2-phenoxyethanol and caffeine for 15 min. This exposure time was set based on a previous study^42^. After the fish was transferred from the preparation tank into the measurement tank, it was restrained in the fixture, as shown in Fig. 3c; needle electrodes were inserted 1–2 mm above the bulbus arteriosus and near the anal region at a depth of approximately 1 mm^44^. After 3 min of rest to reduce the effects of transfer, ECG and gill movements were recorded for 120 s. Data obtained from individuals that survived 72 h after the experiment were included in analysis.

#### Experimental conditions

The room and water temperature were maintained at 27 and 29 $^\circ$C, respectively, during the experiment. The concentration of 2-phenoxyethanol was set at $x_a = 260$ $\mu$L/L and that of caffeine was set at $x_c=0, 1, 50, 100$ mg/L based on the parameters used in the previous heart rate measurement study^32^. The concentration of 2-phenoxyethanol was determined based on preliminary experiments. We found that 15 min of exposure to 260 $\mu$L/L 2-phenoxyethanol immobilized the adult zebrafish without causing loss of ventilation. The ECG and ventilatory signals of five individuals were measured at each caffeine concentration (Supplementary Information). The frame rate of the high-speed camera was $f_c = 100$ fps, and the sampling frequency for the ECG was $f_s = {1}{kHz}$.

### Experiments under free-swimming conditions

#### The measurement system

**Figure 5:**
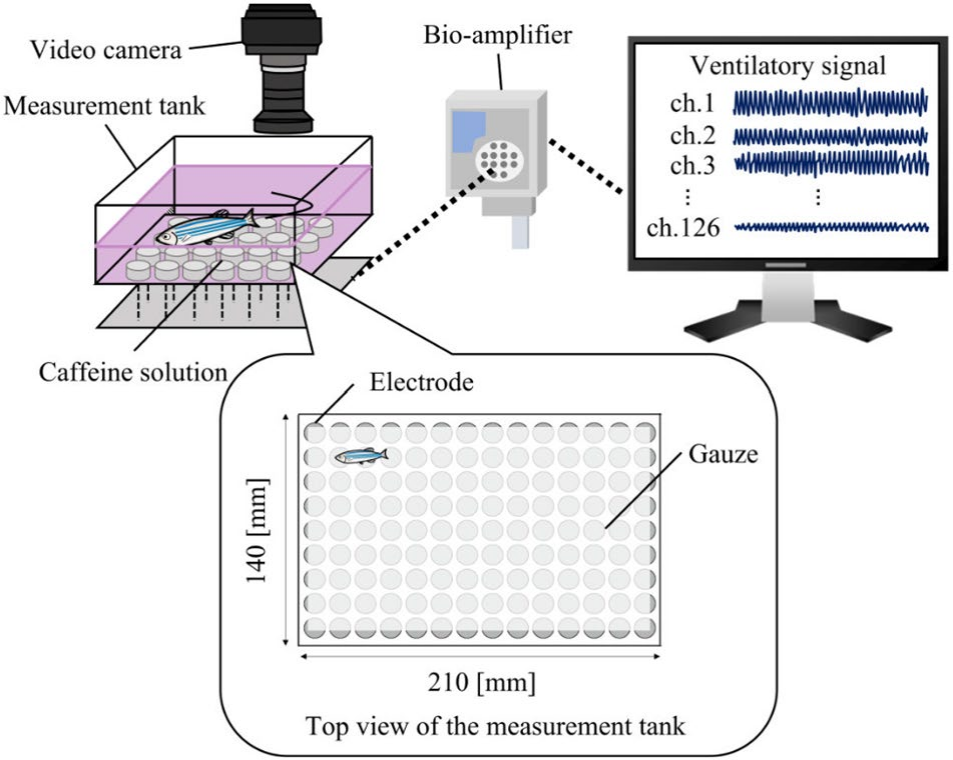
Measurement equipment for experiment under free-swimming conditions.

Figure 5 shows the measurement system for the free-swimming experiment. The measurement system consisted of a measurement tank, video camera, and bioamplifier. The measurement tank was a rectangular double-bottomed structure (210 (W)$\times$140 (H)$\times$65 (W) mm) with 126 Ag-AgCl electrodes placed at the upper bottom and reference and ground electrodes were placed at the lower bottom. The bioelectric signals measured from the electrodes were sampled at $f_k$ Hz using a digital bioamplifier (EEG-1200, Nihon Kohden, Tokyo, Japan). A video camera was installed above the measurement tank to capture the swimming fish at $f_c^{f}$ fps. The position of the fish was tracked using a markerless pose estimation software, DeepLabcut^33^, to select an electrode closest to the fish position in a time interval of *e* s. The signal measured at the selected electrode was used to analyze ventilation.

#### Experimental protocol

**Figure 6:**
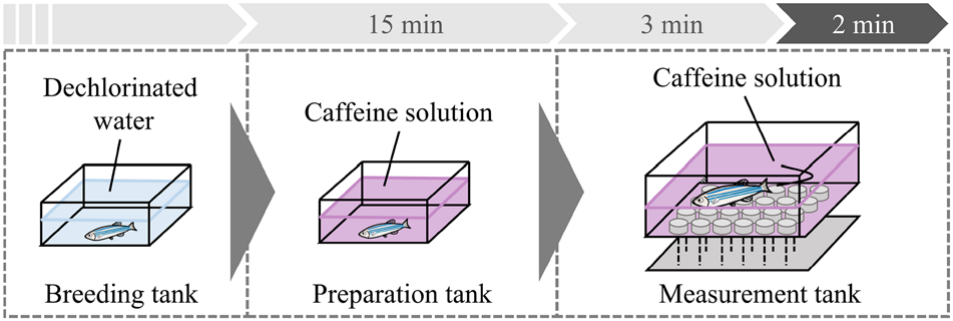
Experimental protocol for the free-swimming conditions.

A mixed solution of dechlorinated water and caffeine was prepared at 400 and 800 mL and placed in the preparation and measurement tanks, respectively. The caffeine concentration was set at $x_c$ mg/L. The caffeine exposure procedure is illustrated in Fig. 6. An individual was randomly selected following the same procedure described in the restraint conditions. The selected individual was transferred from the breeding tank to the preparation tank and exposed to caffeine for 15 min. The individual was then transferred from the preparation tank to the measurement tank. After 3 min of rest to reduce the effects of transfer, ventilatory signals were measured for 120 s. Meanwhile, the position of the fish was recorded using a video camera. Data obtained from individuals that survived 72 h after the experiment were included in analysis.

#### Experimental conditions

Room and water temperatures were maintained between 27 $^\circ$C and 29 $^\circ$C. Caffeine concentrations were set at $x_c=0, 1, 50$, and 100 mg/L, and five individuals were monitored for each caffeine concentration (Supplementary Information S2). The signal measurement electrode was selected at intervals of $e = 5$ s. The frame rate of the video camera was $f_c^{f} = 29.97$ fps, and the sampling frequency of the ventilatory signals was $f_k = {500}{Hz}$.

### Analysis

To analyze the ventilatory response and cardiac activity, the ventilatory frequency and heart rate, as well as the ventilatory interval and heartbeat coefficients of variation, based on heart rate variability analysis^19,20,45^, were calculated from the measured ventilatory signals and ECG. The measured data were then fitted using a linear mixed model for statistical analysis.

**Figure 7:**
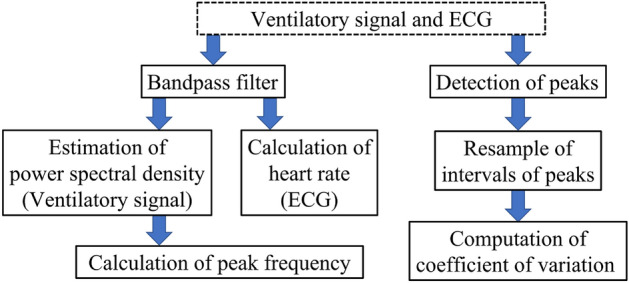
The procedure used to calculate the indices to evaluate ventilation and heart rate.

The analysis method is illustrated in Fig. 7. First, each signal was filtered using an *l*-order Butterworth bandpass filter. The low and high cutoff frequencies (ventilatory signals: $f_{low}^{r}$ and $f_{high}^{r}$, ECG: $f_{low}^{h}$, $f_{high}^{h}$) were set based on the values reported in previous studies^27,44^.

Each filtered time-series signal was divided into *r* s segments, and the amplitudes were standardized to a normal distribution with a mean of 1 and a variance of 0. The peak frequency of ventilation (ventilatory frequency) was calculated by estimating the power spectral density of the ventilatory signals at each time segment (*r* s) using Burg’s method applied to a *p*-order autoregressive model (AR). Heartbeats per second (heart rate) were calculated by detecting the peak number of R waves in each *r* s segment of the ECG.

The coefficients of variation for the ventilatory interval and heartbeat were calculated by detecting the peaks of the corresponding waveform and obtaining the intervals of the detected peaks. For peak detection, the minimum and minimum peak interval thresholds were defined to eliminate false detections. For the ventilatory signals, the thresholds of the minimum and minimum peak intervals were denoted as $\theta _p^{r}$ and $\theta _I^{r}$, respectively. For the ECG, the thresholds of the minimum and minimum peak intervals were denoted as $\theta _p^{h}$ and $\theta _I^{h}$, respectively. Finally, the detected peak interval was resampled at 10Hz.

A linear mixed model was used to address the effect of caffeine on ventilation and cardiac activity, considering the random effects of intra- and inter-individual differences. To this end, the following eight candidate linear mixed models were fitted to the measured data and the best model was selected based on the AIC criterion.

1 $$\begin{aligned} F_j= & {} \beta _1C+\beta _0, \end{aligned}$$

2 $$\begin{aligned} F_j= & {} \beta _1C+\left( \beta _0+\beta _{0,j}\right) , \end{aligned}$$

3 $$\begin{aligned} F_j= & {} \beta _1C+\left( \beta _0+\beta _{0,C}\right) , \end{aligned}$$

4 $$\begin{aligned} F_j= & {} \beta _1C+\left( \beta _0+\beta _{0,j}+\beta _{0,C}\right) , \end{aligned}$$

5 $$\begin{aligned} F_j= & {} \beta _1C+\left( \beta _0+\beta _{0,C,j}\right) , \end{aligned}$$

6 $$\begin{aligned} F_j= & {} \beta _1C+\left( \beta _0+\beta _{0,C}+\beta _{0,C,j}\right) , \end{aligned}$$

7 $$\begin{aligned} F_j= & {} \beta _1C+\left( \beta _0+\beta _{0,j}+\beta _{0,C,j}\right) , \end{aligned}$$

8 $$\begin{aligned} F_j= & {} \beta _1C+\left( \beta _0+\beta _{0,C}+\beta _{0,j}+\beta _{0,C,j}\right) , \end{aligned}$$

where $F_j$ represents a physiological index (heart rate, ventilatory frequency, coefficient of variation of the heartbeat, and coefficient of variation for the ventilatory interval) for the *j*-th individual, $C=\log (x_c+1)$ is the logarithmic caffeine concentration, and $\beta _0, \beta _1$ are the fixed effects of the intercept and slope, respectively. The terms $\beta _{0,C}$, $\beta _{0,j}$, and $\beta _{0,C,j}$ are the random effects of the intercept depending on the subscription, where *C* represents the concentration and the *j*-th individual. These equations did not assume individually-dependent random effects of caffeine, since an individual only experienced one caffeine concentration.

### Analysis conditions

The Butterworth filter order was set to $l=3$, and the ventilatory signal cutoff frequency was set to $f_{low}^{r}={1}{Hz}, f_{high}^{r}={10}{Hz}$. The ECG cutoff frequency was set to $f_{low}^{h}={5}{Hz}, f_{high}^{h}={120}{Hz}$. The order of the AR model was determined using the AIC at each $r={5s}$ segment in the range of $p=10\sim 50$. The time-series data of the ventilatory and heartbeat were divided into segments of $v={5s}$ to calculate the coefficient of variation. The thresholds for the minimum and minimum peak intervals were set to $\theta _p^{r}=0.5, \theta _p^{h}=0.1$ and $\theta _I^{r} = {0.1s}, \theta _I^{h} = {0.2s}$, respectively.

## Supplementary Information

Supplementary Information.

## Data Availability

Source data are publicly available on GitHub (https://www.github.com/yukiharada23/datasets-ventilatory-signals-and-ECG-.git).
