# Supplementary material for: Pharmacological effects of caffeine on ventilation in adult zebrafish under free-swimming conditions
Source: Sci Rep. 2022 Oct 21;12:17649. doi: 10.1038/s41598-022-22681-4 (PMC9587047; doi:10.1038/s41598-022-22681-4)
Supplement: Supplementary file 1 — Supplementary Information. [file 41598_2022_22681_MOESM1_ESM.pdf]

Pharmacological Effects of Caffeine on the Ventilation in Adult Zebrafish Under Free-swimming Conditions

Yuki Harada[1], Zu Soh[1, *], Shin Wakitani[1], Masayuki Yoshida[2, *], and Toshio Tsuji[1, *]

[1] Graduate School of Advanced Science and Engineering, Hiroshima University, Higashi-Hiroshima, 739-8527, Japan
[2] Graduate School of Integrated Sciences for Life, Hiroshima University, Higashi-Hiroshima, 739-8527, Japan
[*] sozu@bsys.hiroshima-u.ac.jp (ZS), yosidam@hiroshima-u.ac.jp (MY) tsuji-c@bsys.hiroshima-u.ac.jp (TT)

Supplemental Information

S1. Estimation of individual number

We used a simulation-based linear mixed model power analysis to estimate the sample sizes for experiments[1]. This analysis employed peak frequencies of ventilation measured under free-swimming conditions described in the main text. Figure S1 shows the power curve. According to the figure, the power to detect an assumed slope of 0.2 for logarithmic caffeine concentration exceeds 80% for five individuals and five repeated measures at each concentration. Because traditionally, 80% power is considered adequate[2], we decided to select five individuals for each concentration.

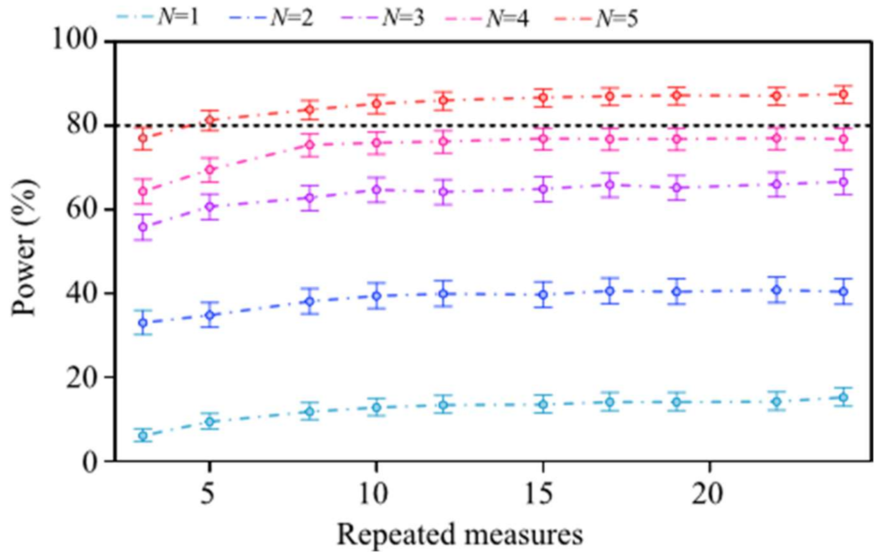

Figure. S1 Result of power analysis.

The *x*-axis denotes the number of repeated measures, the *y*-axis represents the power of detecting an assumed slope of 0.2 for logarithmic caffeine concentration. Four levels of caffeine concentrations (0, 1, 50, and 100 mg/L) were used. The colors of dotted lines indicate the number of individuals (N = 1–5).

S2. The relationships between the ventilatory signal and gill movement under restraint conditions

We compared the frequencies of gill movements and ventilatory signals. The ventilatory signal and the gill movement were measured under restraint conditions. The measurement system used in the restraint condition was modified to simultaneously measure ventilatory signals and gill movements. Figure. S2(a) shows the measurement system consisting of a fixture, measurement tank, high-speed camera, bioamplifier, and three electrodes. The fixture was made up of a conductive urethane sponge hollowed in the shape of a fish and wrapped in transparent vinyl to hold the fish in place. The fixture was fixed to the bottom of the measurement tank using hook and loop fasteners. An Ag-AgCl electrode was placed on the fixture close to the gill to measure the ventilatory signal, and two Ag-AgCl electrodes were placed on the bottom of the tank as the reference electrodes. The measured ventilatory signal was sampled at f_s Hz using a digital bioamplifier (EEG-1200, Nihon Kohden, Tokyo, Japan). A high-speed camera (Ace USB3.0, Basler AG, Ahrensburg, Germany) was installed above the measurement tank to capture images of the zebrafish head at f_c fps to measure gill movement.

The gill movements were measured using the same procedure as described in the main text. Polygonal regions of interest (ROIs) were set around the gill area of the captured video image, as shown in Fig. S2(b). The mean brightness in the ROI was calculated for each frame, and the obtained time-series waveform was defined as the gill movement.

Figure S3 illustrates the experimental procedure. A mixture of dechlorinated water and 2-phenoxyethanol (for anesthesia) was prepared. 400 mL of the mixture was added to both the preparation and the measurement tanks. The concentration of 2-phenoxyethanol was x_a μL/L. A randomly chosen individual was transferred from the breeding tank to the preparation tank and exposed to 2-phenoxyethanol for 15 min. After the fish was transferred from the preparation tank into the measurement tank, it was restrained in the fixture, as shown in Fig. S2(a). Finally, the ventilatory signals and gill movements were recorded for 120 s.

The correlation between peak frequencies of gill movements and that of ventilatory signals was assessed. First, each signal was filtered using an l-order Butterworth bandpass filter. The low and high cutoff frequencies (f_{low}, f_{high}) were set based on the values reported in a previous study[3]. Each filtered time-series signal was divided into r s segments, and the amplitudes were standardized to a normal distribution with a mean of 1 and a variance of 0. The peak frequencies were calculated by estimating the power spectral density of each signal at each time segment (r s) using the Burg's method applied to a p-order autoregressive model (AR).

During the experiment, the room and water temperature were maintained at 27 and 29℃, respectively. The concentration of 2-phenoxyethanol was set at x_a = 260 μL/L. The ventilatory signal and gill movement of one individual were measured. The frame rate of the high-speed camera was f_c = 100 fps, and the sampling frequency of the ventilatory signals was f_s = 1 kHz. The order of the Butterworth

filter was set to l = 3, and the cutoff frequency of the ventilatory signal and gill movement was set to f_{low} = 1 Hz, f_{high} = 10 Hz. The order of the AR model was determined using the AIC at each r = 5 s segment in the range of p = 10 – 50.

Figure S4 shows the scatter plot between the peak frequencies of the ventilatory signals and gill movements. This analysis demonstrated that the peak frequencies are highly correlated (r = 0.981, $p <$ 0.001), indicating correspondence between the ventilatory signals and gill movements.

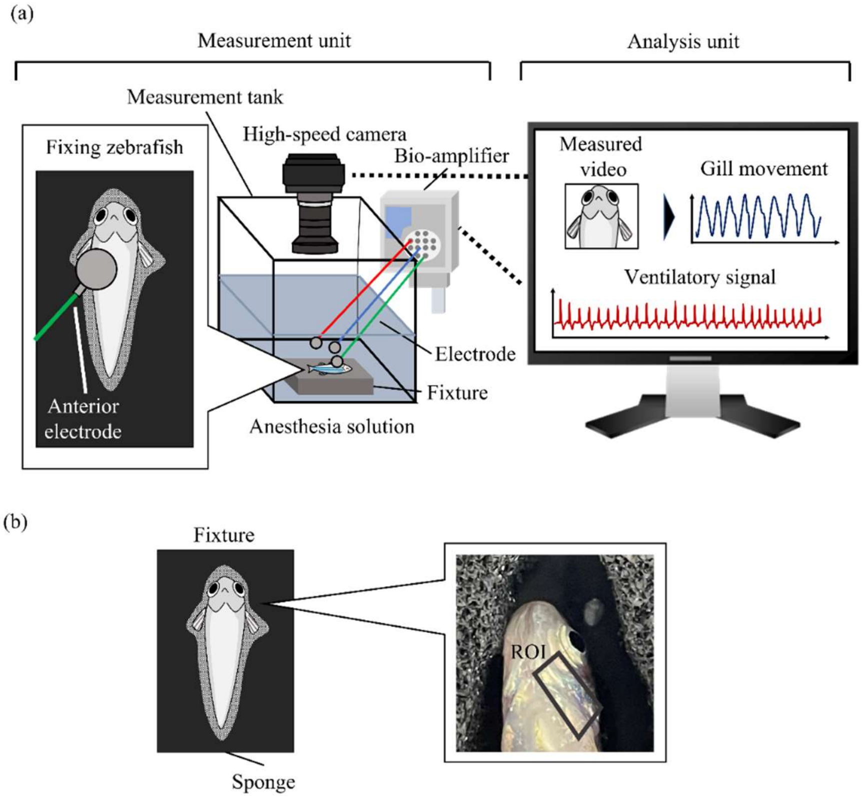

Figure S2. Measurement equipment for experiment. (a) Schematic diagram of measurement and analysis units. (b) Region of interest (ROI) around the gill.

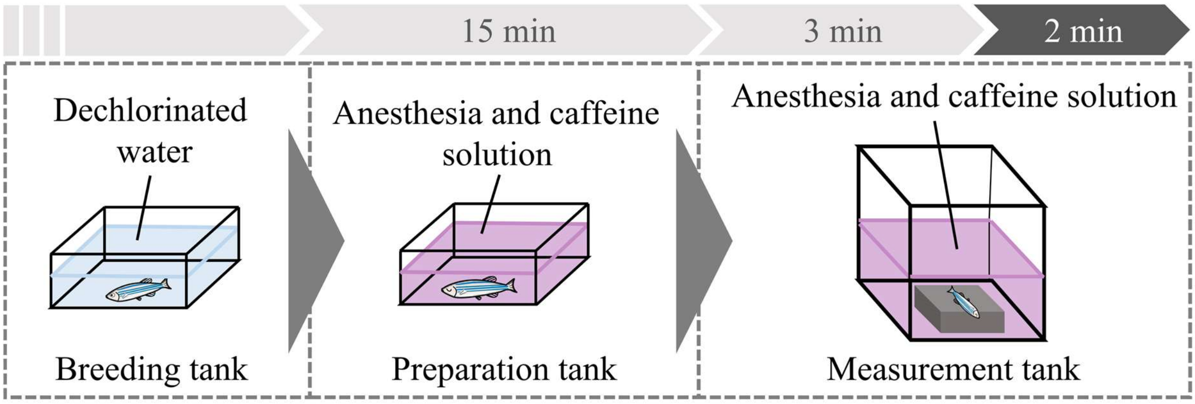

Figure S3. Experimental protocol.

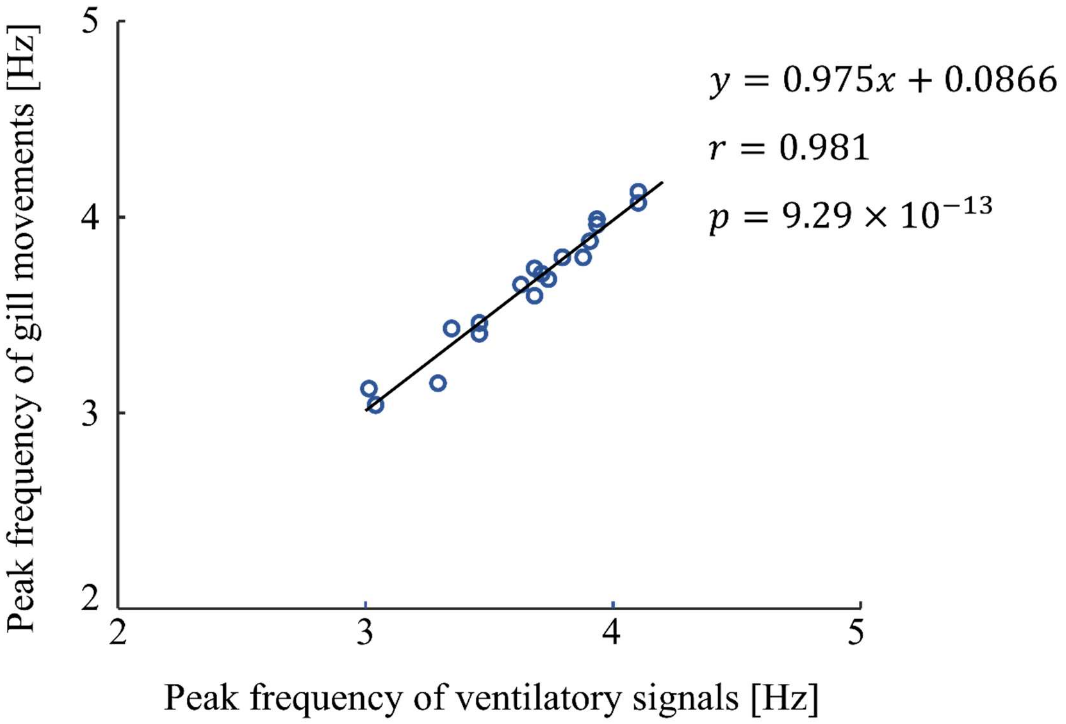

Figure S4. The scatter plot of the peak frequencies of gill movements and ventilatory signals.

References

1. Green P, MacLeod CJ (2016). "simr: an R package for power analysis of generalised linear mixed models by simulation." *Methods in Ecology and Evolution*, 7(4), 493–498.

2. Cohen, J. (1992). Statistical power analysis. Current directions in psychological science, 1(3), 98-101.

3. Soh, Z., Matsuno, M., Yoshida, M., Furui, A. & Tsuji, T. Measurement of emotional states of zebrafish through integrated analysis of motion and respiration using bioelectric signals. Sci. reports. 11, 1–11 (2021).
